# 

**Published:** 1782

**Authors:** 


### PROMOTED.

*March* 30. Mr. —— Graham to be Surgeon's Mate to the garriſon at Gibraltar.

*April* 9. Mr. Andrew Graves, hoſpital-mate, to be Surgeon to the 53d regiment of foot, in the room of Mr. George Currie. Mr. H. M. Menzies, to be Surgeon to the 84th regiment of foot, in the room of Mr. Alex. Daviſon.—11. P. M. Aug. Brouſſonnet, of Montpelier, M. D. Matthew Guthrie, M. D. phyſician at Peterſburgh, and David Pitcairn, M. B. to be Fellows of the Royal Society.—18. Mr. John Pearſon to be additional Surgeon to the Lock Hoſpital. —25. John Gunning, Eſq; to be Fellow of the Royal Society.—27. Mr. Alexander Barr to be Surgeon to the 22d regiment of dragoons.

*May* 11. Mr. William Graham to be Surgeon to the 103d regiment of foot.— 16. Mr. Vincent

Wood to be Surgeon to the forces in Jerſey.— 18. Mr. James Muir, hoſpital-mate, to be Surgeon to the 3d regiment of foot.—29. Mr. John Mallet, of the 45th of foot, to be Surgeon to the 11th regiment of dragoons. Mr. William Hatchet, hoſpital-mate, to be Surgeon to the 45th regiment of foot.—31. Dr. James Hervey to be Phyſician to the Lock Hoſpital, in the room of Sir Noah Thomas, Knt. who has reſigned.

*June* 22. Mr. John Ruſh to be Surgeon to the 2d troop of horſe grenadier guards. Dr. William Sinclair to be Surgeon to a corps of foot to ſerve in Newfoundland.—25. Mr. Patrick Lindſay to be Surgeon to the 45th regiment of foot, in the room of Mr. Hatchet.

### D I E D.

*January*. Mr. Robert Boyes, ſurgeon to the 15th regiment of foot, killed at the ſiege of Brimſtone-hill, in the iſland of St. Chriſtopher.

*March* 25. At Cuckfield in Suſſex, Mr. Chatfield, apothecary.—29. In Queen-ſtreet, Weſtminſter, Dr. Thomas Wilbraham, Fellow of the College of Phyſicians and R. S. and formerly phyſician to the Weſtminſter Infirmary.

*April* 21. At Folkſtone in Kent, Mr. T. Nickalls,

Nickalls, furgeon and apothecary.—29. At Hackney, Dr. Thomas Dawfon, Member of the College of Phyficians, and formerly Phyfician to the London Hofpital.

*May.* At Wymondham, Norfolk, Mr. James Carver, furgeon. At Coventry, Mr. Bennet, apothecary.—2. At York, Mr. Anthony Hubback, apothecary.—5. At Bowling, near Bradford, Yorkfhire, Mr. John Whitaker, apothecary.—13. Daniel Charles Solander, M. D. in the Univerfity of Upfal, LL. D. in that of Oxford, Fellow of the Royal Societies of London, Stockholm, Upfal, &c. and one of the Under Librarians of the Britifh Mufeum. This celebrated Naturalift was born in Sweden May 28 1736. His death was occafioned by a fit of apoplexy, with which he was attacked on the 9th of May while in converfation, with his ufual cheerfulnefs, at his friend Sir Jofeph Banks's in Soho-fquare. On diffection, a confiderable quantity of coagulated blood was found in the lateral ventricles of the brain.—At Nottingham, aged 32, Mr. John Hollis, furgeon.—16. Of a pulmonary confumption, at Reading in Berkfhire, on his way to Ireland, Dr. Dennis Ryan, late Affiftant Phyfician to the Military Hofpital in Jamaica, and author of two ingenious Effays, publifhed in former numbers of our Journal. He was born in 1746 at New Park near Cafhel in Ireland, and ftudied Phyfic at Edinburgh and Paris. He graduated at Rheims in 1779, and the year following fet out for Jamaica, with evident fymptoms of incipient phthifis. The ill ftate of his health obliged him to return to England in 1781. He has left be hind him accounts of two or three interefting cafes, which perhaps will appear in fome future number of our work; and a Latin tranflation of Dr. Duncan's *Medical Cafes*, which is in the hands of Profeffor Hahn at Leyden, who has undertaken to get it printed.—At Saffron Walden in Effex, Dr. Brown, aged 89.—17. At Bath, Mr. Bufh, apothecary.—19. In Upper Brook-ftreet, Weftminfter, aged 82, Mr. Buller, apothecary. At Bath, Mr. John Donne, furgeon.—23. At Dewfbury, Yorkfhire, Mr. Battye, furgeon.

*June* 4. At Berlin, aged 50 years, Dr. Zinnendorf, Firft Phyfician to the Pruffian army.—5. In London, Mr. Robert Duckenfield, furgeon to the 2d troop of horfe grenadier guards. —12. At Maidftone, Kent, aged 72 years, Mr. William Waller, apothecary. At Liverpool, aged 82 years, Mr. Edward Livefley, formerly a furgeon and apothecary in that town.—13. Mr. Samuel Ball Sherfton, furgeon to his Majefty's hofpital fhip the Orford, at Sheernefs.

SEC-

### SECTION IV.

### Quarterly Catalogue.

1. THE Valetudinarians Companion, or observations on air, exercise, and regimen, with the medical properties of the sea and mineral waters of Brighthelmston. By *Loftus Wood, M. D.* Physician to the Misericordia General Dispensary. 8vo. *Watts,* London, 1782. 82 pages, 1s. 6d.

2. A Description of the Influenza; with its distinction and method of cure. By *R. Hamilton, M. D.* and Fellow of the Royal Medicinal Society of Edinburgh. 8vo. *Johnson,* London, 1782. 31 pages, 1s.

3. Tentamen Medicum Inaugurale quædam de vino complectens. Auctore *Geo. M'Clennachan.* 8vo. Edinb. 1781. p. 34.

4. Dissertatio Inauguralis, quædam de Fermentatione exhibens. Auctore *Gulielm. Leister.* 8vo. Edinb. 1781. p. 37.

5. De Systematis Nervosi Officiis, ejusque conditionibus nonnullis, Tentamen inaugurale. Auctore *Carolo Stuart.* 8vo. Edinb. 1781. p. 58.

6. Dissertatio Inauguralis de Scorbuto. Auctore *Jacobo Home.* 8vo. Edinb. 1781. p. 49.

Tentamen

7. Tentamen Medicum Inaugurale, amplectens quædam de Vasis absorbentibus. Auctore *Joh. Winterbottom.* Edinb. 1781. p. 49.

8. Dissertatio Inauguralis, de Plantarum incrementi Causis. Auctore *Archibaldo Lindsay.* 8vo. Edinb. 1781. p. 43.

9. Dissertatio Medica Inauguralis de Lue Venerea. Auctore *Gulielm. Harrison.* 8vo. Edinb. 1781. p. 58.

Three experiments are related in this Dissertation. In the first, the matter of a chancre was introduced into the Urethra and excited a Gonorrhœa. In the second, the inner surface of the prepuce was inoculated with the matter of a Gonorrhœa, but without producing any effect. In the third, matter from a chancre was mixed with quicksilver that had been previously extinguished with gum arabic, and afterwards introduced by inoculation into the skin in various parts of the body. But this, like the second experiment, produced no effect. The two last experiments, we are told, were repeated several times and always with the same event.

10. Dissertatio Medica Inauguralis de Apoplexia sanguinea. Auctore *Joh. Johnstone.* 8vo. Edinb. 1781. p. 34.

11. Memoirs of the Life and a view of the character character of the late *Dr. John Fothergill.* Drawn up at the desire of the Medical Society of London. By *Gilbert Thompson*, M. D. Member of the Royal College of Physicians, and Secretary to the Society. 8vo. *Cadell*, London, 1782. 45 p.

12. De l'Electricité du corps humain dans l'etat de santé & de maladie. Ouvrage couronné par l'Academie de Lyon, dans lequel on traite de l'electricité de l'atmosphere, de son influence & de ses effets sur l'economie animale, &c. Par *M. l'Abbé Bertholon* de S. Lazare, des Academies Royales des Sciences de Montpellier, Beziers, Lyon, &c. *i. e.* Of the Electricity of the human Body, in health and in sickness. A work crowned by the Academy at Lyons, and which treats of the electricity of the atmosphere, of its influence and effects on the animal œconomy, &c. By the *Abbé Bertholon* of St. Lazarus, &c. 12mo. Paris, 1780. 540 p.

13. *C. F. Wensels* des Chimie, &c. *i. e.* Chemical Essays on Metals, written with a view to ascertain their constituent principles. By *C. F. Wensels*. 4to. Leipsic, 1781.

In a work printed in 1773 the author gave a singular treatise on the nature of gold, and some other metals, but without describing his processes. Mr. Cappel in opposition to that essay maintained maintained that gold did not give the refults mentioned by Mr. Wenfels. The latter has now publifhed his proceffes.

14. Aphorifmos de Cirurgia de Hermann Boerhaave, comentados por fu difcipulo Van Swieten, y traducidos al Caftellano, con las notas de M. Luis. *i. e.* The Chirurgical Aphorifms of Herman Boerhaave, commented on by his difciple Van Swieten, and tranflated into Spanifh, with the notes of M. Louis. By Don *Juan Galifleo y Xiorro*, Profeffor of Phyfic and Member of the Royal Medical Academy at Madrid. 8vo. Madrid. 1779.

15. Arzneykundige Abhandlungen, herausgegeben von dem Collegio der Aerzte in London, *i. e.* Medical Tranfactions publifhed by the College of Phyficians in London. Tranflated from the Englifh by *D. C. Chr. Kraufe*, 8vo. 3 vols. Leipfic, 1778.

16. *Henrici Palmatii Levelingii*, Philof. et Med. Doctor, Anat. Chirurg. et Inftitut. Medic. in Univerfitate Anglipolitana, Prof. Publ. &c. Obfervationes Anatomicæ rariores de Valvula Euftachii et foramine ovali, quas pro gradu Doctoris defendit *Franc. Xavier de Buglioni.* 4to. Anglipoli (Ingolftadt) 1780, with two copper-plates.

17. Differtatio Inauguralis practico-medica de

Mercurii

Mercurii Sublimati Corrosivi in siphylide efficaci tutoque usu, &c. Auctore *G. Wissikally*. 8vo. Vienna, 1780. p. 40.

18. *Schonh. Lud. Fink* de Morbis biliosis anomalis, occasione Epidemiæ cujus historia præmissa est, ab anno 1776 ad 1781 in Comitatu Teclinburgensi observatæ. 8vo. Munster, 1781.

19. Medinisch œkonomische untersuchung der eigenschafter und würkungen eines æchten und verfælschten puders, &c. *i. e.* A medico-œconomical inquiry concerning the properties and effects of pure and adulterated powder, with all the known and some new modes of preparing it. By *Christian Fred. Reuss*, M. D. & Prof. 8vo. Tubinguen, 1781. 96 p.

This is a very learned Dissertation on hair powder. The author inquires into its etymology and history, and describes the deleterious effects of bad, and the properties of good powder. He calculates that 7200 bushels of wheat are annually consumed in this manufacture in a country inhabited by 10,000 persons, if only a thirtieth part of them use it. He points out other substances, besides starch, which may be substituted for powder.

20. Lettere sopra A. Corn. Celso, al celebre Abate Girolamo Tiraboschi. i. e. Letters to the celebrated celebrated Abbé G. Tirabofchi concerning A. Corn. Celfus. 8vo. Rome, 1779. 278 pages.

Thefe letters, which are twelve in number, are the production of the late Dr. Bianconi, whofe death is announced page 101 of our prefent volume.

21. Fafciculus Animadverfionum phyfiologici atque mineralogico-chemici argumenti. Auctore *Carolo Henrico Köftlino*, Phil. et Med. Doct. Acad. Florent. et Societat. Teuton, Jenenf. Membro. 4to. Stuttgard, 1780.

The firft of the three effays contained in this work, is entitled, "Difquifitio Obfervationum "Cl. della Torre de figura molecularum cruoris "fanguinis." Father della Torre, in his experiments on the blood, made ufe of glafs lenfes, or rather globules, by which the diameter of the object was more increafed than by any microfcope hitherto invented. By means of thefe lenfes he difcovered that the red particles of the blood which had generally been fuppofed to be of a globular fhape by phyfiologifts, were in fact flat cylindrical rings, each of which was furnifhed with a little veficle or bladder.—The author of the work before us having had occafion to fee the ingenious father repeat his experiments, fufpected that his opinions relative to the fhape of of thefe particles were not well founded. He has fince repeated the experiments with glaffes of different diameters given to him by father della Torre, and he acknowledges that the refults correfponded pretty exactly with della Torre's account; but he is of opinion that the real appearance of the object is changed by the lens; for when globules of coloured glafs, made fo fmall as to be hardly perceptible to the naked eye, were viewed through della Torre's glaffes, the middle of thefe alfo feemed to be fomewhat tranfparent, and he thinks there is no doubt but that if they could be made fufficiently minute to reprefent the globules of the blood, they would likewife give the appearance of a ring.

The fecond effay is intitled, "Examen mineralogico-chemicum materiei quæ Herculaneum et Pompeios anno Ær. Chrifti 79 fepelivit," and the third relates to the origin of the *Pumex officinalis*.

22. Sammlungen zur Phyfik und Naturgefchichte von einigen Liebhabern diefer Wiffenfchaften, *i. e.* A Collection of tracts on Natural Philofophy and Natural Hiftory. Vol. I. 8vo, Leipfic, 1779, with 8 copper-plates.

This collection contains profeffor Pallas's obfervations on the formation of mountains, and the changes that have happened to the globe;

 Mr.

Mr. Cavallo's treatife of electricity; and feveral tracts from the Philofoph. Tranf. Rozier's Journal, Buffon's hiftory, and other works of reputation.

23. *Ignatii A Born*, Equ. Acad. Imp. Nat. Cur. Petropolit. Londin. &c. Sodalis, Index rerum naturalium Mufei Cæfarei Vindobonenfis. Pars I. Teftacea. 8vo. Vienna 1778, with one copper-plate.

24. Teftacea Mufei Cæfarei Vindobonenfis, quæ juffu Mariæ Therefiæ Auguftæ difpofuit et defcripfit *Ignatius A Born*, Eques, &c. Folio. Vienna, 1780, with 18 coloured engravings.

25. Auctarium ad *Alb. Halleri* Elementa Phyfiologiæ Corporis humani excerptum ex nova editione et adaptatum veteri. 4to. Leipfic, 1780.

This Supplement to Haller's Phyfiology is intended to accommodate thofe who are in poffeffion of the Quarto edition of that work. It is to be completed in eight numbers, one for each volume. Three numbers and part of a fourth are already publifhed; thefe include all the additions in the eight volumes (all that are as yet publifhed) of the octavo edition.

26. Chemifch Phyfifche Schriften; *i. e.* Chemico-phyfical Effays. By *Francis Charles Achard*,

Pro-

Professor of Chemistry, and Member of the Academy of Sciences at Berlin, &c. 8vo. Berlin, 1780.

We have here twenty essays, the greater part of which relate to Philosophical Chemistry. In the fifth our author endeavours to prove that the teeth are constantly growing. He kept two squirrels upon soft food, which could require no mastication, and after a certain time he observed that their teeth were considerably lengthened; they continued to grow till at length the animals were unable to open their mouths, and died through inanition.

The ninth essay relates to some experiments on the elastic gum, and a cheap method of making surgical instruments with it, by dissolving it in oil of turpentine, and precipitating it with spirit of wine. The gum, we are told, then appears in the form of a thick mucilage, which resumes its elasticity upon being dried in the air.

In the twelfth we have an account of a palsy cured by electricity; and in the thirteenth essay the author treats of the incubation of eggs by electricity.

27. Von der Würkung des Mohnsaffts in der lustseuche, &c. *i. e.* Of the effects of opium in the venereal disease. To which are added some observations relative to the diseases and natural history hiſtory of North America. By *John David Schæpff*, M. D. Phyſician to the troops of Anſpach-Bayreuth. With a preface by the editor, *J. Delius*, M. D. and Prof. 8vo. Erlangen, 1781.

A young Engliſhman was ſo afflicted with the lues, that the pain ariſing from his ulcers deprived him entirely of reſt. In this ſtate, opium mitigated the pain, brought on a better digeſtion of the ulcers, and was the chief means of procuring a cure which before had been deſpaired of.—The author remarks, that the ſame remedy has ſince been given in other ſimilar caſes, and with ſucceſs. Its effects are very ſatisfactorily accounted for by the editor in his preface, who ſuppoſes that it acts in the ſame manner in venereal ulceration as belladonna, cicuta, &c. do in cancers.

The author gives an account of ſome comparative experiments with different patients, in which he obſerves, that thoſe who were treated with mercury ſuffered more and much longer than thoſe who took opium. The work cloſes with ſome remarks on the climate and ſeaſons of North America, and their influence on the health of the natives and ſtrangers.

28. Diſſertatio Medica de ſenſibilitate oſſium mormorbosa, quam Preside *Adolpho Murray* sustinuit *Laurentius Brandelius*, M. A. 4to. Upsal, 1781.

Professor Murray attributes the sensibility of the bones to the nerves, which he contends pass even to the medulla. He mentions several cases to prove that the bones often become sensible when in a carious state.

There are several parts of the animal body which, tho' insensible in an healthy state, are rendered more or less sensible by disease. Our author has remarked this change of disposition in tendons, cellular membrane, and bones, and likewise that the sensibility of the nerves is gradually increased. He attributes the difficulty of discovering the nerves in the bones to this, that their extremities lose their cylindrical shape, and, becoming flattened, are with difficulty distinguished from the cellular texture.

The compression of the nervous fibrillæ in a sound state of the bones, he supposes to be the cause of the insensibility of the latter, and that hence they acquire feeling in proportion as they become carious and spongy.

29. Dissertatio Inauguralis Medica, exponens vulnerum cum deperditione substantiæ Medelam. Auctore *Ottone Philippo Wiedenfeld.* 4to. Gottingæ, 1781.

30. Dis-

30. Differtatio Medica Inauguralis de Hepatitide. Auctore *Jofepho Sigifbert Miquel.* 4to. Nancy, 1781.

31. De limitanda laude librorum medicorum practicorum ufui populari deftinatorum. Auctore *J. A. Murray*, M. D. & Prof. 4to. Gottingen, 1779. p. 32.

Since the appearance of M. Tiffot's *Avis au Peuple*, France and Germany (we may add England too) have been deluged with popular treatifes on phyfic. This has given rife to the prefent work.

32. Differtation fur les avantages de l'allaitement des enfans par leurs meres; ouvrage qui a été couronné par la Faculté de Medecine de Paris, dans fa feance publique le 9 Decembre 1779. *i. e.* Differtation on the advantages refulting from mothers fuckling their children; being the work that obtained the prize given by the Faculty of Phyfic of Paris at their public meeting, December 9, 1779. By M. Landais, M. D. Phyfician at Effarts in Poitou. 8vo. Paris, 1781. 55 pages.

